# Delaying circadian sleep phase under ultradian light cycle causes time-of-day-dependent alteration of cognition and mood

**DOI:** 10.1038/s41598-023-44931-9

**Published:** 2023-11-20

**Authors:** Fanny Fuchs, Ludivine Robin-Choteau, Aline Schneider, Laurence Hugueny, Dominique Ciocca, Tsvetan Serchov, Patrice Bourgin

**Affiliations:** 1 Institute for Cellular and Integrative Neurosciences (INCI)-UPR 3212-CNRS/University of Strasbourg, 8 allée du Général Rouvillois, 67000 Strasbourg, France; 2 Sleep Disorders Center and CIRCSom (International Research Center for ChronoSomnology), Strasbourg University Hospital, 1 place de l’Hôpital, 67000 Strasbourg, France; 3 European Center for Diabetes Studies (CEED), Strasbourg, France; 4 Chronobiotron-UMS3415-CNRS/University of Strasbourg, Strasbourg, France

**Keywords:** Circadian rhythms and sleep, Circadian regulation, Sleep, Cognitive neuroscience, Neuroscience, Learning and memory, Short-term memory, Spatial memory

## Abstract

Light exerts powerful and pervasive effects on physiology and behaviour. These effects can be indirect, through clock synchronization and phase adjustment of circadian rhythms, or direct, independent of the circadian process. Exposure to light at inappropriate times, as commonly experienced in today’s society, leads to increased prevalence of circadian, sleep and mood disorders as well as cognitive impairments. In mice, exposure to an ultradian 3.5 h light/3.5 h dark cycle (T7) for several days has been shown to impair behaviour through direct, non-circadian, photic effects, a claim we challenge here. We first confirmed that T7 cycle induces a lengthening of the circadian period resulting in a day by day phase-delay of both activity and sleep rhythms. Spatial novelty preference test performed at different circadian time points in mice housed under T7 cycle demonstrated that cognitive deficit was restrained to the subjective night. Mice under the same condition also showed a modification of stress-induced despair-like behaviour in the forced swim test. Therefore, our data demonstrate that ultradian light cycles cause time-of-day-dependent alteration of cognition and mood through clock period lengthening delaying circadian sleep phase, and not through a direct photic influence. These results are of critical importance for the clinical applications of light therapy in the medical field and for today’s society to establish lighting recommendations for shift work, schools, hospitals and homes.

## Introduction

Light plays a fundamental role in regulating physiology and behaviour. During the past decades, growing use of light-emitting devices such as smartphones, computer screens or tablets, as well as exposure to light pollution in general, lead us to be exposed to light at inappropriate times of day. This extensive and non-physiological use of artificial light entails an increased prevalence of circadian rhythms disorders, insomnia, daytime somnolence, mood alteration and cognitive and attentional deficits^1–4^. Moreover, a significant and growing part of the population is engaged in “atypical” work schedules, such as shift- or night-work^5^ with exposure to artificial light at aberrant time, again leading to serious health consequences such as sleep and alertness disturbances, cognitive alteration, metabolic disorders or even a predisposition to cancer^6–9^. Particularly, sleepiness, fatigue, and decreased performances are more present when employees work rotating shifts, compared to night workers^10^. On the opposite, light is also widely known to have therapeutic effects, especially when applied to mood disorders^11–13^. Light exerts two kinds of effects, indirect through clock resynchronization and phase shifting of circadian rhythms, and direct by sending signals to structures in the brain that control the behaviour^14, 15^, so its physiological or therapeutic influence on behaviour is partly due to its role as a circadian clock regulator, but also to its direct effects. It is therefore difficult to disentangle the mechanisms by which light modulates cognitive functioning and mood in humans.

In rodents, which are nocturnal animals, short pulses of darkness acutely promote wake behaviour whereas short periods of light exposure induce sleep^16^. During several decades, such effects were referred as “positive and negative masking” respectively^17^, but since a few years the terminology of “direct light effects” has been adopted to point out the process that actively imposes itself on the expression of sleep and waking^15^. In mice, ultradian light–dark cycles can be used to investigate this direct photic regulation of behaviour with light pulses administered at various times of day. In this regard, LeGates, et al.^18^ used an ultradian light–dark cycle composed of alternating 3.5 h light/3.5 h dark (T7) to determine the direct influence of aberrant light exposure on mood and cognitive function, as they considered that the circadian system remained functional in this condition. In their paper, they observed detrimental effects of T7 cycle on behaviour, so concluded that light directly impairs mood and memory. We hypothesized that this ultradian cycle affects circadian rhythmicity and sleep and waking distribution. Therefore, we sought at complementary experiments using the same light–dark regimen to re-evaluate whether light directly impairs mood and memory or whether this aberrant light alters the circadian system, subsequently affecting behaviour in a circadian fashion.

In this study, we first evaluated the impact of T7 light–dark cycle on general activity and sleep to confirm that T7 induces a chronic phase-shift of both parameters, with a lengthening of the circadian period, resulting in a complete phase inversion after 12 days of ultradian cycle exposure. Then, the analysis of T7 effects on memory and mood (spatial novelty preference test and forced swim test), in comparison to standard T24 cycle (12 h light/12 h dark), showed that the modification of behaviours observed under T7 cycle were circadian-time dependent. Therefore, aberrant light (exposures at inappropriate times) does not have direct deleterious effect on cognition and mood, but rather induces non-physiological modulation of circadian clock, resulting in circadian variation of cognitive and mood functions and driving to altered behaviors at certain times of day.

## Results

### T7 ultradian cycle induces a lengthening of the period of activity and sleep–wake rhythms

**Figure 1:**
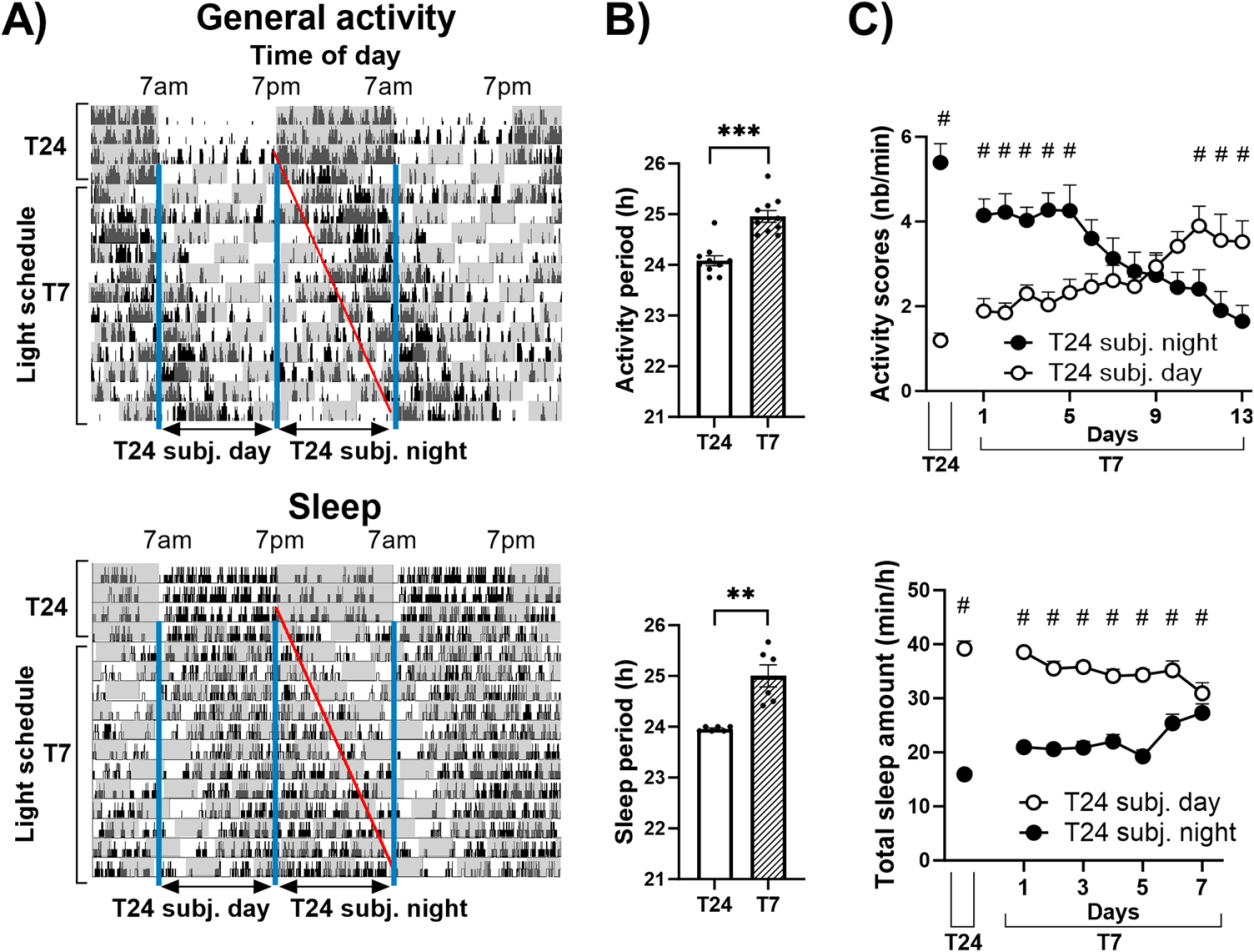
T7 cycle induces a lengthening of the circadian period of general activity and sleep–wake rhythms. (**A**) Representative double-plotted actogram (upper part) and double-plotted diagram of sleep (lower part) from one mouse under T24 and T7 cycles (grey areas delineate the dark exposures). Black tick marks represent activity scores (upper part) or NREM (lower part, lower ticks) and REM (higher ticks) sleep. Red line shows the phase-shift observed under T7 cycle. Subjective day and night during T7 cycle correspond respectively to light and dark 12 h-exposures during T24 cycle. (**B**) The period τ of activity (upper part) and sleep rhythm (lower part) was significantly increased under T7 cycle compared to the T24 one (***p < 0.005; **p < 0.01). (**C**) Activity scores (mean number of beam crossings per minute, upper part), greater during nights than days under T24 cycle, were progressively and significantly shifted under T7 cycle, to become higher during subjective days (day vs night under T24: ^#^p < 0.005; subjective days *vs* subjective nights for days 1–5 and 11–13 of T7 cycle: ^#^p < 0.05 at least). Inversely, total (NREM + REM) sleep (mean duration per hour, lower panel), which occurred essentially during the day under T24 cycle, was also shifted under T7 cycle (day *vs* night under T24: NK, ^#^p < 0.005; decreasing difference between subjective days and nights in T7 cycle: NK, ^#^p < 0.005 for days 1–6 and p < 0.05 for day 7). Data are represented as mean + SEM. Actimetry: n = 10; Sleep: n = 6. *Subj* subjective.

By measuring general activity and sleep of mice under two undisturbed baseline days (12 h light/12 h dark called T24 cycle) and 13 days of T7 cycle, we observed a day by day delay of the activity phase when exposed to T7 cycle (representative examples of actogram and sleep diagram in Fig. 1A). This phase-shift was due to a period lengthening to approximately 25 h of both activity (Fig. 1B, upper part; 24.96 ± 0.12) and sleep rhythms (Fig. 1B, lower part: 25.00 ± 0.22) in T7 cycle, which significantly differed from the classical 24 h period in T24 cycle (actimetry: 24.08 ± 0.10; sleep: 23.96 ± 0.02; Paired sample *t*-test, activity: t_(9)_ = 5.92; ***p = 0.0002; sleep: t_(5)_ = 4.65; **p = 0.0056). To quantify this progressive shift, we averaged activity counts and total (NREM + REM) sleep amount during each subjective day and night periods under T7 cycle, corresponding to previous light and dark 12 h-exposures under T24 cycle. General activity (Fig. 1C, upper part), which was higher during the night under T24 cycle, progressively shifted during T7 cycle to be mainly expressed during the subjective day, with a total phase inversion after 12 days of T7 cycle exposure (two-way ANOVA (day × phase interaction): F_(13,117)_ = 19.19; p = 0.0000; post-hoc test: day vs night under T24: NK, ^#^p < 0.005; subjective days *vs* subjective nights for days 1–5 and 11–13 of T7 cycle: NK, ^#^p < 0.05 at least). Total sleep amounts (Fig. 1C, lower part), which were higher during the day under T24 cycle, was also shifted under T7 cycle, as observed during the first 7 days of exposure (two-way ANOVA (day × phase interaction): F_(7,35)_ = 11.40; p < 0.005; post-hoc test: day vs night under T24: NK, ^$^p < 0.005; decreasing difference between subjective days and nights in T7 cycle: NK, ^$^p < 0.005 for days 1–6 and p < 0.05 for day 7).

**Figure 2:**
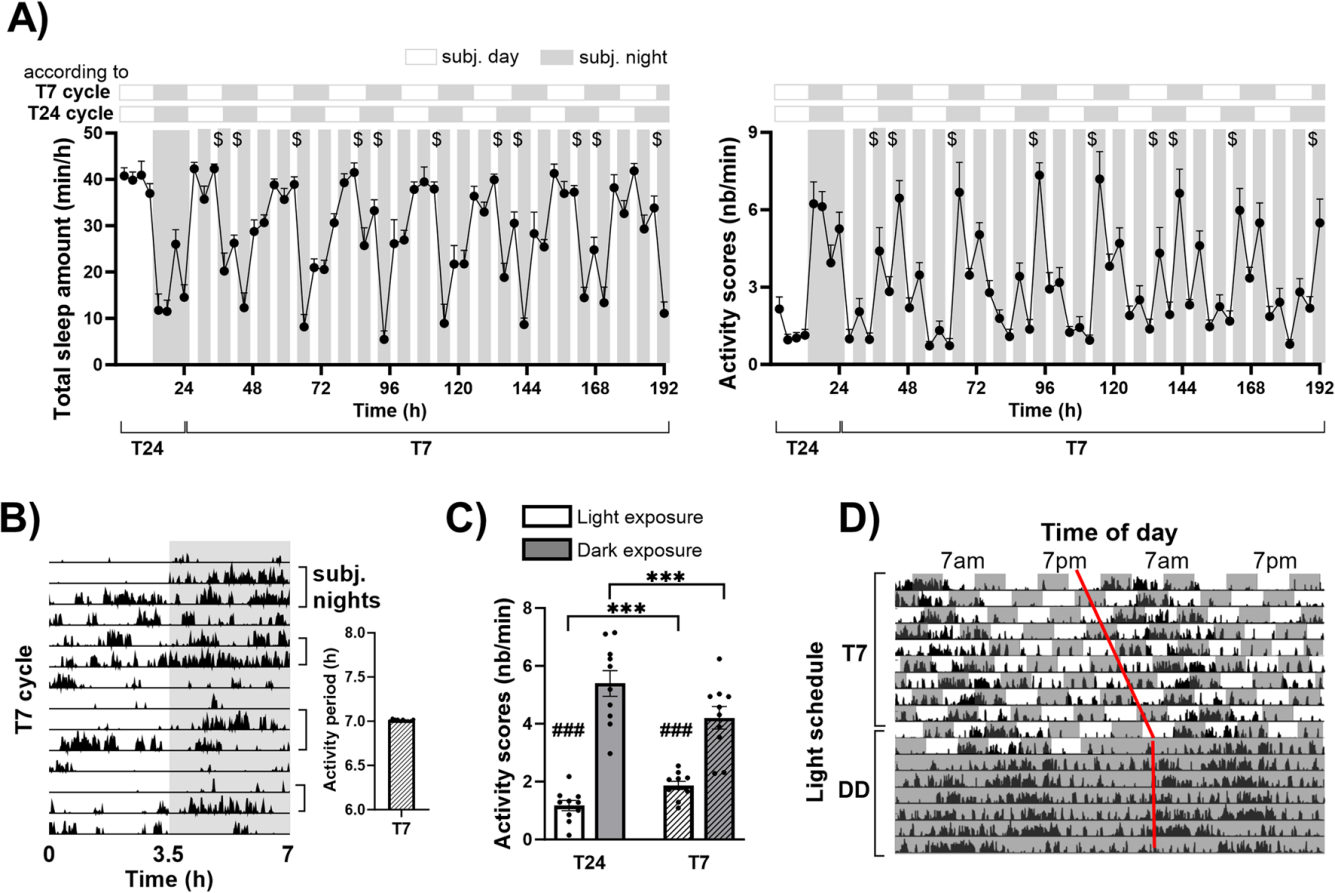
The modification of activity and sleep distribution under T7 cycle relies on both direct effects of light on behaviour and light-induced modulation of circadian rhythmicity. (**A**) Light pulses directly promoted sleep (left panel) and inhibited activity (right panel) whereas the inverse effect was observed for darkness exposures. These effects were greater during subjective nights (sleep amounts: ^$^p < 0.05 for comparisons 2–3, 6, 9–10, 13, 16–17, 20–21 and 24; activity counts: ^$^p < 0.05 for comparisons 2–3, 6, 0, 13, 16–17, 20 and 24). Grey areas represent dark exposures. T24 subjective day-night alternation (lower horizontal bars) correspond to the 12-h periods of day and night under T24. T7 subjective days and nights (upper horizontal bars) correspond to the 12.5-h periods of theoretical days and night under T7, considering the 1-h period lengthening. (**B**) Representative simple-plotted actogram (left panel) from one mouse under T7 cycles per 7 h-periods (grey areas delineate the dark exposures, black tick marks represent activity scores and brackets represent subjective nights). A significant short-component (7.011 h ± 0.004) period of activity was observed under T7 cycle for the 10 studied mice (right panel). (**C**) Activity scores averaged for light and dark exposures under T24 and T7 cycles were higher during dark exposures in both conditions, but to a lesser extent under T7 than T24 cycle (light *vs* dark exposures under both T24 and T7 cycles: ^###^p < 0.005; T24 vs T7 cycle for both light and dark exposures: ***p < 0.005). (**D**) Representative double-plotted actogram from one mouse for the last 10 days of T7 cycle exposure and the subsequent 7 days of DD exposure (grey areas delineate the dark exposures). Black tick marks represent activity scores. Red line shows the phase-shift observed under T7 cycle and the new phase of activity onset expressed under DD exposure. Data are represented as mean + SEM. Actimetry: n = 10; Sleep: n = 6. *Subj* subjective.

To examine more precisely the direct influence of light on activity and sleep distribution, we compared sleep amounts (Fig. 2A, left part) and activity scores (Fig. 2A, right part) between consecutive light and dark exposures under T7 cycle. Light exerted direct sleep-promoting and activity-inhibiting effects whereas darkness promoted active waking. Indeed, sleep amounts were higher during light pulses compared to dark exposures, the difference between consecutive light and dark pulses being significant for almost half of the light–dark comparisons (ANOVA, F_(1,47)_ = 17.64; p = 0.0000; NK ^$^p < 0.05 at least for comparisons 2–3, 6, 9–10, 13, 16–17, 20–21 and 24). Inversely, activity scores were often higher during darkness exposures than light pulses (ANOVA, F_(1,47)_ = 14.06; p = 0.0000; NK ^$^p < 0.05 at least for comparisons 2–3, 6, 10, 13, 16–17, 20 and 24). During T7 cycle, in addition to the lengthened circadian period of 25 h, we also observed a significant short-period component of 7 h in all animals (7.011 ± 0.004; Fig. 2B), corresponding to the direct effects of light on activity (or masking effects of light). Activity scores were then averaged for actual light and dark exposures under T24 and T7 cycles (Fig. 2C). In both conditions, animals were more active during dark than light exposures; however, this difference was milder under T7 cycle compared to T24 cycle, an effect due to both an increase of activity during light periods and a decrease during dark exposures (two-way ANOVA (cycle × light exposure): F_(1,9)_ = 62.37; p < 0,005; post-hoc test : light vs dark exposures both under T24 and T7 cycles: NK, ^###^ p < 0.005; T24 vs T7 cycle for both light and dark exposures: NK, *** p < 0.005). This was explained by the maintenance of a circadian rhythm of activity inducing a global increase of activity during subjective nights, whatever the actual lighting condition. After T7 cycle exposure, animals were maintained in constant darkness for 7 days in order to observe the expression of their endogenous period of activity. As illustrated in Fig. 2D, this endogenous period was very close to 24 h (23.99 ± 0.13; Supplementary Fig. 1) and the activity onset corresponded to the one observed at the end of T7 cycle exposure (12-h phase delayed from initial circadian phase under T24).

Therefore, our data supported the idea that T7 ultradian light–dark cycle influenced general activity and sleep by inducing a non-physiological lengthening of the circadian period, leading to a complete inversion of the circadian phase after 12 days of T7 exposure, as well as through direct sleep promoting effects of light. Given these results, our aim was to assess whether T7 cycle induces memory and mood alteration and whether those resulted from direct effects of light and/or from these modifications of circadian organization.

### Light-induced day by day phase-delay drives to circadian time-dependent cognitive and mood alterations

**Figure 3:**
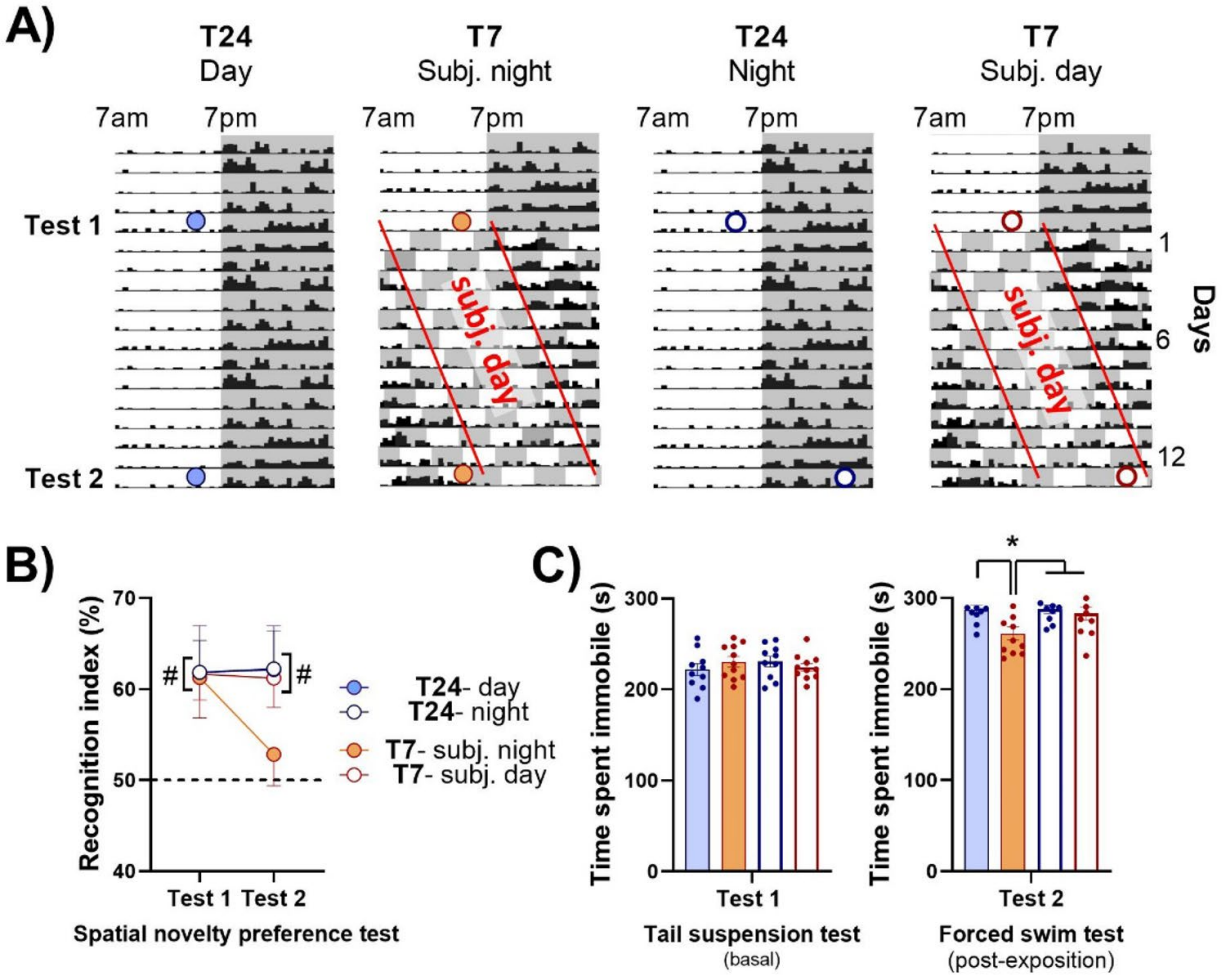
T7 cycle induces circadian variations of cognition and mood. (**A**) Experimental schedule of each of the four groups of animals is illustrated on schematic actograms. Coloured rounds indicate behavioral tests performed 13 days apart. Red lines show the phase-shift observed under T7 cycle: T7 subjective days represent the theoretical periods of day under T7 that are delayed by 12 h after 12 days of exposure, which correspond to day-to-day 1-h period lengthening. (**B**) During spatial novelty preference task, recognition indices [novel arm/(novel + familiar arms) × 100] for the first test were significantly higher than chance level (50%) for all 4 groups of animals. For the second test performed 12 days later, the “T7-subj. night” group only had poorer performances, not significantly different from chance level (^#^p < 0.05 at least. “T24-day”: blue disk; n = 11; “T7-subj. night”: orange disk; n = 12; “T24-night”: blue circle; n = 11; “T7-subj. day”: orange circle; n = 13). (**C**) All four groups of mice spent similar amount of time immobile during the tail suspension test performed during the day after T24 cycle exposure (left part). During forced swim test (right part), the group “T7-subj. night” was the only one to spend less time immobile than the others (*p < 0.05 between “T7-subj. night and the three other groups. “T24-day”, n = 12; “T7-subj. day, n = 11; “T24-night”, n = 10; “T7-subj. night”, n = 11). Data are represented as mean + SEM. *Subj* subjective.

The spatial novelty preference task in T-maze was used to evaluate the effect of T7 cycle exposure on memory performances. Each mouse was exposed to one arm of the maze during the sample phase (5 min). After an inter-trial interval of 1 min, the animal was free to visit the entire maze and the recognition index reflected the ability of the animal to remember the arm visited before. To estimate the respective influence of the direct photic effects and those from the circadian drive, and given the phase inversion of circadian rhythms observed after 12 days of T7 cycle exposure, we tested 4 groups of animals at specific and appropriate time points (Fig. 3A, Test 2): animals exposed to T24 were tested at the end of the day (“T24-day”; ZT9) or end of the night (“T24-night”; ZT21), and those exposed to T7 at the end of subjective night (“T7-subj. night”, corresponding to the same time than “T24-day” group) or end of subjective day (“T7-subj. day”, corresponding to the same circadian time than “T24-night” group). We first ensured that the groups had similar baseline performances, by conducting the test at the end of the day under baseline T24 exposure (Fig. 3A; Test 1). Our results showed that all four groups, when tested during the day under T24 cycle (Fig. 3B; Test 1), were able to remember the arm visited during the sample phase, showing a recognition index significantly higher than chance level (50%; Two-tailed *t*-test: Group “T24-day”: t_(10)_ = 2.30, ^#^p = 0.044; Group “T7-subj. night”: t_(11)_ = 2.53, ^#^p = 0.028; Group “T24-night”: t_(10)_ = 3.41, ^#^p = 0.007; Group “T7-subj. day”: t_(12)_ = 4.06, ^#^p = 0.0016). The T24 animals evaluated 12 days later (Fig. 3B; Test 2), in “T24-day” and “T24-night” groups, were still able to remember the arm visited during the sample phase (Group “T24-day”: t_(10)_ = 2.53, ^#^p = 0.030; Group “T24-night”: t_(10)_ = 2.93, ^#^p = 0.015), therefore revealing no “test–re-test” effect, and no circadian variation of short-term memory. On the contrary, animals exposed to T7 cycle showed deficiency to recognize the familiar arm when tested during subjective night, with a recognition index that did not differ from 50% (t_(11)_ = 0.82, p = 0.43). This deficit did not rely on a decrease of spontaneous exploration of the animals in the maze, the distance travelled during both sampling and test phases of Tests 1 and 2 being similar between “T24-day” and “T7-subj. night” groups (Supplementary Fig. 2). This one time point evaluation does not allow to conclude that aberrant light impairs learning through direct effects independent of the circadian system, as general activity and sleep were phase-inversed after 12 days of T7 cycle. Therefore, it was important to evaluate memory performances when assessed at the opposite of the phase (“T7-subj. day”). In this condition, our results showed that memory abilities were unaffected, with a recognition index significantly higher than 50% (t_(12)_ = 3.46, ^#^p = 0.0048). Therefore, chronic phase shift-induced memory alteration was dependent on circadian time but did not rely on a direct effect of light. These results were confirmed by applying a 2-h light pulse during the night (at ZT21) (a duration sufficient to exert direct photic effects on behaviour as illustrated by sleep promotion^19, 20^) and testing revealed that animals recognition index remained higher than chance (Supplementary Fig. 3).

To investigate whether the T7 cycle induces mood alteration in a circadian manner or through direct photic effects, we evaluated the behaviour of mice exposed to an aversive and inescapable situation by measuring the immobility time during the tail suspension test (TST) as a basal evaluation, and during the forced swim test (FST) after T24 or T7 cycle exposure according to times points used for memory testing (see previous section; Fig. 3A). All animal groups spent similar amount of time immobile in the TST, under baseline T24 condition (Fig. 3C, left part; ANOVA, F_(1,3)_ = 0.36; p = 0.78). To avoid a test-re test effect, we used the forced swim test 13 days later (Fig. 3C, right part). The impact of 12 days of T7 exposure assessed at the same activity phase (“T7-subj. day”) or at opposite phase (“T7-subj. night”) showed a decrease of time spent immobile when tested during the subjective night specifically. Indeed, the group of mice tested during subjective day presented no modification of their reaction to this aversive situation compared to the ones maintained under T24 cycle, whereas “T7-subj. night” group spent less time immobile than the three other groups (ANOVA, F_(1,3)_ = 3.81; p = 0.017; NK *p < 0.05 between “T7-subj. night” and the three other groups). Therefore, chronic phase-shift induced a modification of mood that was circadian time-dependent but did not rely on a direct effect of light.

## Discussion

In humans, chronic exposure to light at inappropriate times of day can lead to sleep and circadian disorders, cognitive defects and depression. In this regard, the impact of ultradian light–dark cycle, as experienced with shift work, remains poorly understood. Here, we show that ultradian light–dark cycle induces alterations of cognitive and mood functions that are not due to direct photic effects but are relying on a non-physiological regulation of the circadian clock delaying the circadian sleep phase.

First, we confirmed that exposure to such ultradian light cycle, even if it does not induce an abolishment of the circadian function, causes a period lengthening (25-h) leading to a day-by-day phase-delay of circadian rhythms of both general activity and sleep^18, 21^. This period lengthening results in a complete inversion of the circadian phase after 12 days of T7 cycle exposure, and seems particularly non physiological given that the endogenous circadian clock period is lower than 24 h in mice^22^. These effects seem to rely both on direct effects of light on behaviour and on light-induced circadian modulation.

Indeed, we observed under T7 cycle a direct sleep-promoting effect of light and a wake promoting effect of darkness, corresponding respectively to the ancient terminology of negative and positive masking^17^. In addition, under T7 cycle we also found out a short-component 7-h long rhythm of activity, with higher activity scores under darkness and conversely higher amounts of sleep during light pulses. In that respect, according to our data, the repeated alternation of light and dark pulses of short duration was sufficient to modulate activity levels but also sleep–wake distribution throughout the entire T7 cycle exposure. These results are in contradiction to those previously published by Samer Hattar’s group^23^, who showed no effects of light on sleep during T7 cycle (possibly explained by the large age range of mice used in their study; n = 4, from 4 to 12 months), although they described a significant effect on wheel-running activity. Moreover, our findings are coherent with multiple data of the literature that reported the somnogenic effect of light pulses during ultradian cycles of different durations^20, 24–27^. In addition, one can assume that, if the direct effects of light were the only factor to modulate behavior under T7 cycle, activity scores and sleep amounts would rely on real light conditions only (i.e. on 3.5 h-long light and dark pulses), and a progressive circadian desynchronization would be observed due to these direct effects.

In sum, our data highlight that, under T7 cycle, activity and sleep modifications do not depend on direct effects of light only. Indeed, the findings reveal that the ultradian T7 cycle is also able to entrain the circadian system over a lengthened 25-h long period. The maintenance of circadian variations explains that the difference between activity amounts observed under light and dark exposures was milder under T7 cycle than under T24 cycle, given that activity levels remained overall higher during subjective nights than subjective days. In the same way, the greatest light-induced promotion of sleep appeared in the trough of sleep circadian rhythm (corresponding to the T7-subjective night), when the circadian drive favors waking rather than sleep, whereas the effects of light–dark alternation were attenuated during the periods of long-duration of sleep (corresponding to the subjective day). More than a “ceiling effect” of the sleep-promoting direct influence of light, the data suggest that the overall effect relies on the circadian drive getting the upper hand over waking-promoting direct effect of darkness. Last, when animals are exposed to DD after T7 cycle, the endogenous period calculated over 7 days was very close to 24 h, which is higher than classically described endogenous activity period of C57Bl/6 mice^28^, and the circadian phase of activity was delayed by approximately 12 h. This delay corresponds to the 1-h period lengthening observed for the 12 preceding days under T7 cycle and demonstrates that T7 cycle, besides inducing direct light effects, modifies activity and sleep through the entrainment of circadian clocks with longer circadian period^20, 24–27^.

Given the influence of T7 cycle on circadian rhythmicity of activity and sleep, we aimed to assess whether T7 cycle induces memory and mood alteration and whether those resulted from direct effects of light and/or from these modifications of the circadian organization. In the spatial novelty preference task, memory abilities were similar for animals tested during the day or the night under T24 cycle. Circadian modulation of learning and memory with time of day has been widely studied across species ranging from invertebrates to humans but no consensus has been found, since results vary depending on the task, the memory process or the type of memory long-term or short-term, for a review^29^. Though, our data were in accordance with some of the studies that showed no effect of circadian phase on memory abilities, especially when light condition is controlled throughout the behavioural testing^30, 31^, as in our experiments. Animals exposed to T7 cycle showed deficiency to recognize the familiar arm when tested during subjective night. This memory disruption was in accordance with the one observed by Legates et al.^18^ with object recognition and Morris water maze tasks after two weeks of T7 cycle exposure, although the information on the time of testing is missing in their publication. However, given the phase inversion observed after 12 days of T7 cycle, this one time point evaluation is not sufficient to conclude that aberrant light impairs learning through direct effects independent of the circadian system, as assumed by Legates et al.^18^. If memory disruption observed in T7 group was due to a direct effect of light, animal’s performances in T-maze should also be altered when assessed during subjective day. Yet, our results showed that memory abilities were unaffected at this circadian time point. These results, besides the preservation of memory performances observed after 2 h of light exposure at early night under T24, demonstrate that memory impairments observed in mice exposed to ultradian light cycle are not due to direct effects of light, contrary to the assumption of LeGates et al.^18^, but rather rely on non-physiologic regulation of the circadian clock, which is in accordance with human data showing that the non-circadian direct influence of light improves alertness and cognitive performances^1, 32, 33^.

In the same way, the modification of mood-related behaviour induced by exposure to ultradian light–dark cycle is also dependent on circadian timing. Indeed, in the FST, behaviour was modified only during subjective night in mice exposed to T7 cycle, this group showing less time spent immobile compared to the three other groups. These results suggest that prolonged exposure to an ultradian light–dark cycle affects the reaction to stress, dependently of circadian-time, and could particularly modify the ability to cope with an acute inescapable stressor^34, 35^. The decrease of immobility time of the “T7-subj. night” group does not seem to depend on the greater amount of activity during subjective night given that it is not observed during the night under T24 cycle. This time-dependent effect could rely on a circadian rhythm-gated pathway, preferentially conducting light information to brains involved in mood regulation at certain moments of the day/night cycle^36^. Several studies using clock genes mouse mutants have shown circadian periodicity modulations along with mood-related behaviours alterations: especially, mice with a lengthened circadian period of activity showed a decreased immobility time in the FST whereas the inverse was observed in mice showing a shortened circadian period (for a review, see^37^). Then, lengthening the circadian period of activity seems to be associated with behaviours mimicking the one observed in manic state of bipolar patients. Of course, is our case, the use of the FST alone does not allow us to infer the same interpretation. Nevertheless, our results demonstrate that exposure to T7 cycle promotes circadian variations of mood that are not due to a direct effect of light.

Among limitations of the present study, we choose not to monitor corticosterone levels as a previous report showed no difference between T7 and T24 cycles at time points that are close to the ones we used for behavioural evaluations^18^. Additional behavioral evaluations would be useful to assess the effect of T7 cycle exposure on other functions, including anxiety-like behaviors, and to conclude on the potential appearance of manic-like state. Moreover, the same experiments should be performed with female mice in order to evaluate the influence of sex. Finally, it would also be interesting to test the effect of other ultradian light cycles, with various durations of light and dark pulses, on cognition and mood. Nevertheless, the main strength of this study is in its design integrating sleep and circadian data along with behavioral evaluations at different circadian time points. This research design allowed us to disentangle the mechanisms, direct versus indirect (affecting the circadian function), by which ultradian light cycles affect behaviour.

Therefore, clock period lengthening and chronic day by day phase delay promotes the emergence of circadian variations of cognitive functions and mood and drives to memory inability and modifications of strategies adopted in an aversive situation at certain times of day. This study helps to better understand cognitive and mood alterations associated with circadian disorder, especially delayed sleep phase disorder^38^, or non-24 h sleep–wake disorder as well as those induced by shift working^39^. A better understanding of these mechanisms is crucial for improving the management and patient care of circadian disorders, and for optimizing the management of light exposure in today’s society.

## Methods

### Subjects and housing conditions

C57BL/6 adult male mice were obtained from our animal facility, the Chronobiotron (CNRS UMS 3415). They were housed in transparent Makrolon cages (33 cm × 17 cm × 14 cm) in groups of 2–3 in a room with controlled temperature (24 ± 1 °C) and humidity (40 ± 5%) under a 12 h–12 h light–dark cycle (polychromatic white light at 500 lx). Zeitgeber Time (ZT) 0 corresponds to light-on at 7:00 a.m. They had ad libitum access to food and water. Animals used for sleep recording were re-housed singly the day of surgery (Experiment 1), the ones used for memory evaluation were re-housed singly two weeks before the first memory test (Experiment 2), and the ones evaluated in mood tests were maintained in their original group (Experiment 3).

Experimental protocols and animal care were in compliance with European Community Council Directive 2010/63/EU and the current project was approved by the Regional Ethical Committee of Strasbourg for Animal Experimentation and the French Ministry of Higher Education and Research (approval no. 00146.01).

### Experiment 1: influence of T7 cycle on general activity and sleep–wake distribution

#### Light–dark schedule

General activity (actimetry) and sleep (ECoG-EMG) were simultaneously recorded during two undisturbed baseline days under 12 h–12 h light–dark cycle called “T24 cycle”, during 13 days of 3.5 h–3.5 h ultradian light–dark cycle called “T7 cycle” and 7 days of constant darkness (or “dark-dark”, DD) exposure.

#### Actimetry

General activity in their home cage was recorded in 10 mice using actimetry infrared motion detection. Detectors were mounted to the top of each cage and the number of infrared beam detections was collected continuously for 5 min-bins (CAMS, Circadian Activity Monitoring System, INSERM, France). The period (τ) was calculated using ClockLab software (ActiMetrics, Evanston, IL) for T24, T7 and DD exposures. The number of crossings was averaged per minute for objective (T24 cycle) or subjective (T7 cycle) days and nights and for real light and dark exposures (T24 and T7 cycles).

#### Sleep

Six of the 10 mice recorded with actimetry were simultaneously recorded with ECoG-EMG. At the age of 2–3 months, these mice were subjected to surgery protocol for electrodes implantation. Under deep anaesthesia with intraperitoneal injection of ketamine (80 mg/kg) and xylazine (10 mg/kg), animals were implanted with three screws serving as two ECoG and one ground electrodes. Two EMG electrodes were inserted into the neck muscles along the back of the skull. All electrodes were then soldered to a connector and cemented to the skull before the skin was sutured. A minimum of 14 days was given to recover from surgery and to habituate to the recording conditions before any recording began. Signals were recorded using commercial Compumedics hardware (Neuvo 64-512 Channel EEG HD-LTM) and software (Profusion PSG4 Software). ECoG and EMG signals were amplified, filtered, and analog-to-digital converted at 500 Hz for ECoG and 250 Hz for EMG. Sleep stages were manually classified in 10-s epochs according to criteria classically used in mice: non-rapid eye movement (NREM) sleep was distinguished by high-amplitude ECoG dominated by synchronized delta activity (1–4 Hz) and a low EMG signal; REM sleep was defined by a regular low-amplitude theta rhythm (4–12 Hz) and a low EMG signal; wakefulness (W) was characterized by a higher and variable EMG and a low-amplitude ECoG with both slower (delta during drowsiness) and faster (theta during exploratory behaviour) components. ECoG and EMG signals were analysed for the two days of T24 cycle and the first 7 days of T7 cycle. We checked that the phase-shift was maintained for the 13-days period in 4 animals (as illustrated in the sleep diagram exposed in Fig. 1A). The period (τ) was calculated using ClockLab software for T24 and T7 cycles. Total (NREM + REM) sleep duration was calculated over the first 7 days for objective (T24 cycle) or subjective (T7 cycle) days and nights and for individual light and dark exposures (T24 and T7 cycles).

### Experiment 2: influence of T7 cycle on memory performances

#### Spatial novelty preference test

The ability of animals to remember spatial environment was assessed in a separate cohort of experimentally naive animals in a room dedicated to behavioural tests illuminated with 50 lx. Each mouse was placed in this room 10 min before testing for habituation. Spatial novelty preference test relies on the fact that mice explore preferentially novel over familiar environments. The apparatus consisted of a T-maze constructed from grey Plexiglas. Each arm was 24 cm long, 5 cm wide with 16-cm-high walls. Our protocol was based on the one of Bannerman et al.^40^. During the sampling phase, one of the left or right arms was closed, the mouse was placed at the end of the “start arm” and free to explore the start and the other available arm (forced arm) for 5 min (beginning from the time the mouse first left the start arm). Entry into an arm was defined when a mouse placed all four paws into it. Position of forced arm (right or left) was counterbalanced within each experimental group. After an inter-trial interval of 1 min in its home cage, the mouse was returned to the maze for the test phase during which it could freely explore the entire apparatus for 2 min (again beginning from the time the mouse first left the start arm). The amount of time spent in each of the arms of the maze was recorded during both the sampling and test phases. For test phase, we calculated a recognition index [novel arm/(novel + familiar arms) × 100] and compared it to chance level (50%), reflecting the ability to remember the arm free to visit during sampling phase. Given that novelty feature reduces over time, this index was calculated for the first minute of the test only. Apparatus was cleaned with 10% ethanol solution between sampling and test phases and between each mouse.

#### Experiment schedule (Fig. 2A)

At the age of 2–3 months, mice were isolated and placed in a new facility room. After 2 weeks of habituation under T24 cycle, a first test was performed at the end of the day (ZT9). After this first evaluation, the animals were exposed to different light schedules and re-tested after 12 days, forced choice arm being inverted. Animals of the “T24-day” group (n = 11) were continuously exposed to a T24 cycle to evaluate the effect of re-test on memory performances. The influence of T7 cycle on spatial memory capability was assessed in the “T7-subj. night” group (n = 12) which was exposed to T7 cycle between the two memory tests. For the animals of the “T24-night” group (n = 11), continuously exposed to T24 cycle, the second evaluation was performed at the end of the night to evaluate memory abilities at the opposite of the activity phase. Finally, “T7-subj. day” group (n = 13) was exposed to T7 cycle before the second test that was performed at the end of the “subjective day” part of their activity rhythm.

### Experiment 3: influence of T7 cycle on the reaction to an aversive situation

A separate cohort of experimentally naive animals was used and maintained in groups of 2 to 3 animals to avoid the bias due to housing isolation on performances for review^41^. The tests were performed in a room dedicated to behavioural evaluation illuminated with 500 lx. The two tasks were used to assess behavioural response of mice placed in an aversive and inescapable situation.

#### Tail suspension test (TST)

The apparatus consisted of a hook hang on a horizontal wall positioned 30 cm above the floor. Each mouse was hung by its tail on the hook with medical tape. The tail was passed through a hole done in a middle transparent wall placed 20 cm above the floor in order to avoid the mouse to grasp. Recordings of all tests were performed and were manually analysed off line. The time spent immobile was measured throughout the 6 min of the test. The apparatus was cleaned with 10% ethanol solution between each mouse.

#### Forced swim test (FST)

The apparatus consisted of a 2 L transparent plastic cylinder tank filled with water of 23 °C (± 0.5 °C) and 13 cm-high. Firstly developed in rats by Porsolt et al.^42^, this test has been adapted to the mouse more recently^43^. Each mouse was immerged into the water during 6 min with no possibility to escape. After the test, mice were taken off and dried gently, placed under a heat lamp for a few minutes before being taken back to their housing room. A video camera was placed facing the water to record all tests and behaviour was manually analysed off line. The amount of time spent immobile was measured through the 6 min of the test. The mobility was defined as any movements other than those necessary to balance the body and keep the head above the water. Water was changed and temperature was controlled between each mouse.

#### Experiment schedule

At the age of 2–3 months, mice were placed in a new facility room. After 2 weeks of habituation under T24 cycle, the tail suspension test was performed at the end of the day (ZT9). Animals were then exposed to different light schedules and tested after 12 days at two different time points in the forced swim test. The same group conditions were used as for memory evaluation.

### Statistical analysis

All values were expressed as means ± SEM. Statistical analyses were realized using Statistica (Statsoft, Tulsa, OK, USA; version 13). Prior to statistical analyses data assumptions (for example normality and homoscedasticity of the distributions) were verified using Shapiro–Wilk and Levene tests. Paired-sample *t*-test was used to evaluate the influence of light/dark cycle on activity and sleep period. Time-dependent or cycle-dependent changes in activity scores or sleep duration, were assessed with two-way repeated-measures analysis of variance (ANOVA), followed by Newman–Keuls (NK) multiple range tests to determine post-hoc significance. Recognition index of memory test was compared with chance level (50%) using two tailed-sample *t*-test. One-way ANOVA allowed to assess the difference between groups in time spent immobile in TST and FST tests. The threshold for rejecting the null hypothesis was 0.05 throughout.

We confirm that the study is reported in accordance with ARRIVE guidelines. All data are available in the main text or the supplementary materials.

### Supplementary Information

Supplementary Figures.
