# Supplementary material for: Delaying circadian sleep phase under ultradian light cycle causes time-of-day-dependent alteration of cognition and mood
Source: Sci Rep. 2023 Nov 20;13:20313. doi: 10.1038/s41598-023-44931-9 (PMC10662432; doi:10.1038/s41598-023-44931-9)
Supplement: Supplementary file 1 — Supplementary Figures. [file 41598_2023_44931_MOESM1_ESM.docx]

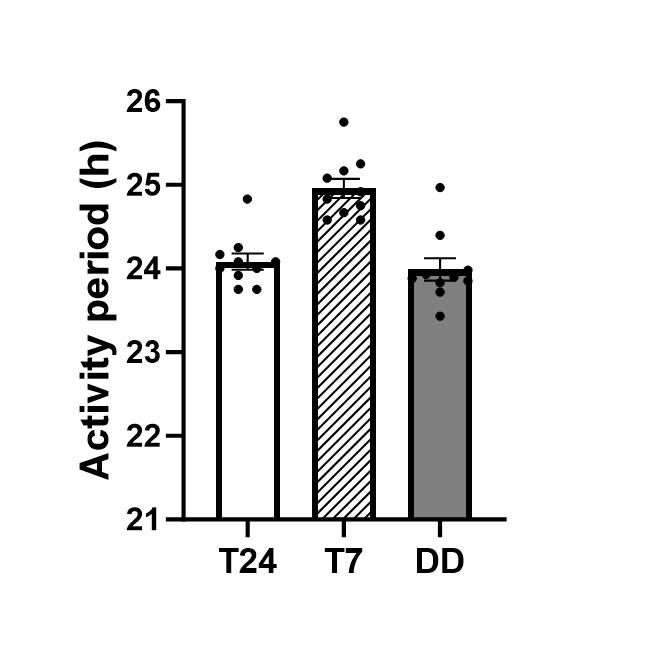


Fig. S1.

**Periods calculated on activity levels over T24 and T7 cycles and over the following 7 days of DD exposure.** Data are represented as mean + SEM. n=10.


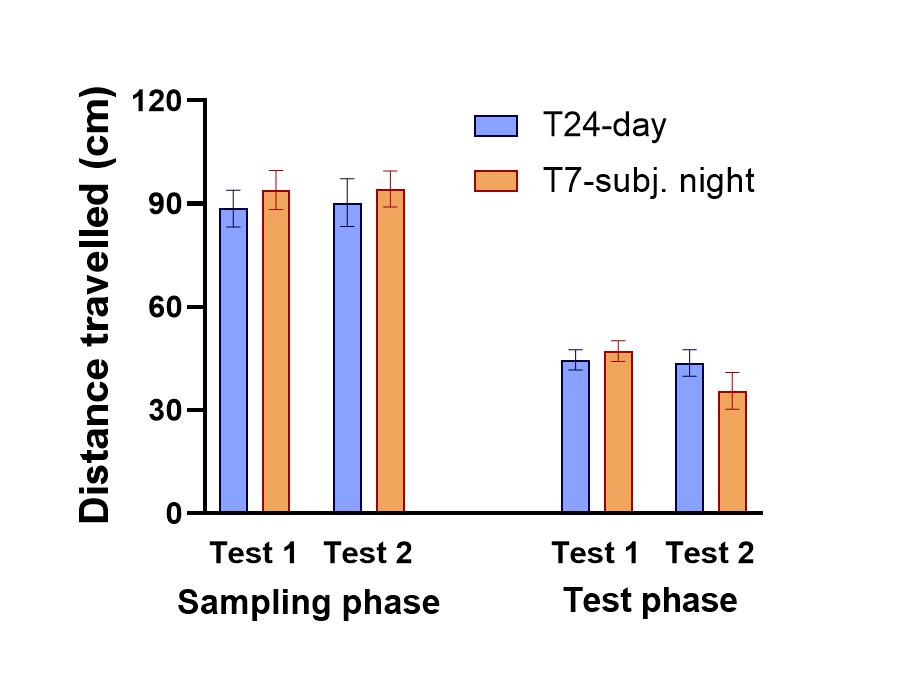


Fig. S2.

**T7 cycle does not influence spontaneous exploration in the T-maze.** Distance travelled during both the sampling and the test phases of Tests 1 and 2 does not differ between “T24-day” and “T7-subj. night” groups (ANOVA (group x test x test phase): non-significant effect of “group” factor, F_(1,1)_=0.03; p=0,86; significant effect of “test phase” factor, F_(1,1)_=371.51; p<0,001; non-significant effect of “test” factor, F_(1,1)_=0.61; p=0.44 ; non-significant interactions). Data are represented as mean + SEM.


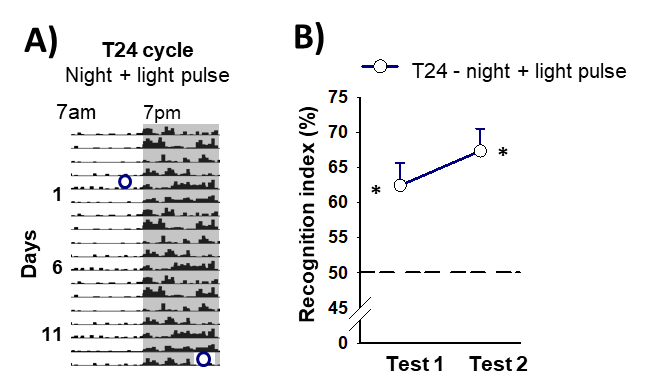


Fig. S3.

**An inappropriate light pulse during the night does not impair spatial memory.** **(A)** Experimental schedule illustrated on a schematic actogram. Coloured rounds indicate T-maze spatial novelty preference tests performed at 13 days interval, the second one being preceded by a 2-h light pulse. **(B)** Recognition indices [novel arm / (novel + familiar arms) x 100] obtained for the first T-maze test (Student t-test : t=3.87, * p<0.005) and the second one (t=5.46, * p<0.005) were both significantly higher than chance level (50%). n=12.
